# Non-invasive detection of pediatric atopic dermatitis based on fecal microbiota and metabolite profiles: a diagnostic approach

**DOI:** 10.3389/fimmu.2026.1836716

**Published:** 2026-06-05

**Authors:** Junsheng Peng, Zifan Li, Wenfeng Wu, Nan Sun, Xianping Yang, Qin Liu, Hongyi Li

**Affiliations:** 1 The Second Clinical College, Guangzhou University of Chinese Medicine, Guangzhou, China; 2 Department of Dermatology, Zhongshan Hospital of Traditional Chinese Medicine Affiliated to Guangzhou University of Chinese Medicine, Zhongshan, China; 3 Department of Dermatology, The Second Affiliated Hospital of Guangzhou University of Chinese Medicine (Guangdong Provincial Hospital of Chinese Medicine), Guangzhou, China

**Keywords:** 16S rRNA sequencing, atopic dermatitis, children, gut microbiota, gut-skin axis, machine learning, metabolomics

## Abstract

**Background:**

Atopic dermatitis (AD) is a common chronic skin inflammation, which affects 15-20% of children worldwide. Gut microbiota and its metabolites are crucial modulators of the “gut-skin axis” in atopic dermatogenesis. However, systematic investigations integrating microbiome and metabolome profiling in mild-to-moderate pediatric AD remain limited.

**Objectives:**

To characterize gut microbiota and metabolic profiles in children with mild-to-moderate AD versus healthy controls, and to identify potential biomarkers and mechanistic pathways involved in disease pathogenesis.

**Methods:**

This single-center case-control study investigated 53 children diagnosed with AD and 16 healthy participants, and collected their fecal samples for microbial and metabonomic analysis.

**Results:**

Mild-moderate pediatric AD patients exhibited significantly increased gut microbial richness and distinct β-diversity compared to controls (PERMANOVA, R²=0.025, *P* = 0.017). Bacteroidota was enriched while Actinomycetota was depleted in AD patients (*P* < 0.05). At genus level, *Parabacteroides* and *Klebsiella* increased, whereas *Bifidobacterium* decreased in AD. Species-level analysis revealed enrichment of *bacteroides_plebeius*, *bacteroides_thetaiotaomicron*, *bacteroides_xylanisolvens*, and *parabacteroides_merdae* in AD. A combined biomarker panel (Bacteroidota, Parabacteroides, and four key species) demonstrated promising exploratory diagnostic potential (AUC = 0.941, accuracy 84.6%), although these results require external validation in larger independent cohorts. Spearman analysis showed correlations between gut microbiome and clinical severity indicators. Thermodesulfobacteriota, Actinomycetota, *Bifidobacterium*, and specific ruminococcus strains positively correlated with the severity of AD. Metabolomics identified 68 differentially accumulated metabolites, primarily involved in lipid metabolism and nucleotide metabolism. Bacteroides species showed significant positive correlations with isovaleric acid levels in microbiota-metabolite analyses.

**Conclusion:**

Mild-to-moderate pediatric AD is characterized by distinct gut microbiota dysbiosis and metabolic alterations involving lipid metabolism. Cross-sectionally identified microbial features show exploratory associations with AD status, but causal inference is not possible. These hypothesis-generating findings support further investigation of the gut-skin axis in AD development and provide a rationale for future interventional studies targeting the microbiome and metabolome.

## 1 Introduction

Atopic dermatitis (AD) is a chronic skin inflammation featured by intense itching and impaired skin barrier (1). As one of the most prevalent skin diseases, AD impacts about 15%-20% of children around the world (2). Severe itching and episodic eczema in pediatric AD patients detrimentally impact quality of life for both children and their families (3). The development of AD is intricate, influenced by genetics, impaired skin barriers, immune disturbances, and environmental factors. Notably, a predominant T helper 2 (Th2) immune response, with elevated levels of interleukin (IL)-4, IL-13, and IL-31, are essential for triggering inflammation and itching (1). Biologics targeting the IL-4/IL-13 pathway have represented major therapeutic advances for moderate-to-severe (1). Despite these advances, significant limitations and unmet needs persist in the management of AD, particularly in pediatric patients. A substantial proportion of children exhibit inadequate response or non-response to dupilumab and similar IL-4/IL-13 inhibitors, often accompanied by residual symptoms, persistent pruritus, or treatment discontinuation due to adverse effects such as conjunctivitis or injection-site reactions (4, 5). Approved biologic options for children remain limited. Selective IL-13 inhibitors such as tralokinumab and lebrikizumab are typically restricted to adolescents aged 12 years and older, resulting in fewer targeted therapeutic choices for school-aged or preschool children, especially those with specific disease subtypes or suboptimal responses to dupilumab (6, 7). Achieving sustained long-term control, durable remission, and complete resolution of refractory pruritus or residual lesions continues to pose substantial challenges. These gaps highlight the need to optimize existing treatment regimens, explore additional pathways, and develop novel agents to provide more comprehensive, personalized, and durable management for the pediatric population.

In recent years, studies have paid special attention to the involvement of gut microbiota and metabolites in the development of AD (8). Intestine is the most important immune organ in our body, and there are countless microorganisms living in it. These bacteria will carry out various metabolic activities and produce many useful molecules, such as short-chain fatty acids (SCFAs) and tryptophan derivatives (9, 10). These compounds modulate immune system development and functionality. Research has confirmed that the gut microbiome can influence the immune homeostasis of distant skin organs through the “gut-skin axis” (11). When the function of the intestinal barrier is impaired or the microbial community is disrupted, bacterial metabolites can enter the circulatory system, thereby regulating systemic immune responses and subsequently affecting skin inflammation (11).

Recent investigations into connections between gut microbiota and AD have yielded significant findings. Several studies indicated a lower diversity of beneficial bifidobacteria in the gut microbiomes of those with AD. Gut barrier compromise permits endotoxin entry into the bloodstream, provoking inflammatory responses (12, 13). And microbial metabolites mediate immune homeostasis. For instance, SCFAs and tryptophan derivatives promote regulatory T-cell differentiation and upregulate anti-inflammatory cytokines, counteracting Th2-skewed immune response central to AD (11, 14, 15). These metabolites also exert direct effects on skin physiology; upon systemic circulation, they modulate keratinocyte function and support epidermal barrier integrity (16–18). Furthermore, maternal-infant gut microbiota dynamics influence early-life immune programming, with dysbiosis in mother-offspring pairs correlating with pediatric AD development (19, 20). In preclinical models, interventions aimed at restoring the balance of microorganisms (such as fecal microbiota transplantation) have been shown to improve AD by normalizing the gut microbiome, increasing SCFA levels, and re-balancing the Th1/Th2 immune response (21–23). These mechanisms underscore the gut microbiota and its metabolites as pivotal regulators of systemic immunity and skin homeostasis in AD pathogenesis.

However, existing research has mostly focused on severe AD or adult patients, with insufficient attention paid to the majority of children with mild to moderate AD. Moreover, most studies are limited to the analysis of microbial composition, lacking in-depth exploration of microbial function. In particular, microbial metabolites, which serve as a critical link between the microbiota and host immunity, have received very limited systematic study. Currently, non-invasive diagnostic approaches for pediatric AD rely primarily on clinical severity scores, which are inherently subjective and dependent on examiner experience. Invasive methods such as serum IgE testing and skin prick tests require blood draws or skin punctures, causing discomfort and anxiety in children, and provide limited mechanistic insight into the gut-skin axis. There is an urgent need for objective, non-invasive, and repeatable biomarkers that can capture underlying pathophysiological processes beyond clinical assessment. Fecal sampling offers a distinct advantage. It is completely non-invasive, painless, easily repeatable, and directly captures gut microbiota and metabolite profiles that reflect systemic immune and metabolic states via the gut-skin axis.

To address this research gap, this study utilizes 16S rRNA sequencing and untargeted metabolomics to characterize gut microbial and metabolic profiles in children with mild-to-moderate AD, aiming to identify potential non-invasive biomarkers and elucidate mechanistic pathways. We hypothesize that these patients demonstrate distinct microbial community compositions and metabolomic signatures compared to healthy controls.

## 2 Materials and methods

### 2.1 Study population and fecal sample collection

This study recruited 53 children (2–12 years old) diagnosed with AD from the Guangdong Provincial Hospital of Chinese Medicine from March 2024 to March 2025, and 16 healthy controls (HC) from the community. Disease severity was assessed by using the Eczema Area and Severity Index (EASI), Investigator’s Global Assessment (IGA), Patient-Oriented Eczema Measure (POEM), Dermatitis Family Impact (DFI), and Children’s Dermatology Life Quality Index (CDLQI). Exclusion criteria for all participants included (1): use of systemic antibiotics, probiotics, prebiotics, or corticosteroids within one month prior to sampling; (2) diagnosis of other chronic inflammatory, autoimmune, or metabolic diseases; (3) recent acute gastrointestinal illness; (4) special dietary regimens. This study was approved by the Ethics Committee of Guangdong Provincial Hospital of Chinese Medicine (Approval No. BF2022-225). A formal priori power analysis was not performed, as this was designed as an exploratory single-center study. Sample size was primarily determined by the number of consecutive eligible participants recruited during the study period.

Participants provided fresh stool samples collected in sterile containers. Approximately 200 mg from each stool’s core was divided into three 2 mL cryogenic vials using sterile spoons. Samples were iced and delivered to the lab within 2 hours, then stored at -80 °C until 16S rRNA sequencing and metabolomic analysis.

### 2.2 Gut microbiota profiling

We extracted microbial community DNA using the HiPure Soil DNA Extraction Kit (Magen, China) and assessed its quality via 1% agarose gel electrophoresis and NanoDrop 2000 UV-vis spectrophotometry (Thermo Scientific, USA). The V3–V4 region of the 16S rRNA gene was amplified with primers 341F and 806R under standard cycling conditions using Q5^®^ High-Fidelity DNA Polymerase (New England Biolabs, USA). Post-amplification, PCR products were verified on 2% agarose gels, purified with AMPure XP Beads (Beckman, USA), quantified with Qubit 3.0, and prepared into libraries using the Illumina DNA Prep Kit. Libraries were qualified by Real-Time PCR and sequenced on a Novaseq 6000 platform with PE250 mode.

### 2.3 Fecal metabolomic analysis

Sample was homogenized in methanol:acetonitrile:water (2:2:1) using MP homogenizer, followed by ultrasonication, incubation, and centrifugation. After collection, we dried the supernatant in vacuum, then freeze-dried, and then stored it at -80 °C. When it is time for analysis, we re-dissolve the sample with 1:1 mixture of acetonitrile and water, fully stir and centrifuge, and finally put the supernatant into the mass spectrometer for detection.

Chromatography on a Vanquish UHPLC system with a HILIC column was conducted at 25 °C and 0.5 mL/min flow rate. Mobile phases were water with ammonium acetate/hydroxide and acetonitrile. The gradient program was: 0–12 min, 95-95% B; 0.5–7 min, 95-65% B; 7–8 min, 65-40% B; 8–9 min, 40% B; 9-9.1 min, 40-95% B; 9.1–12 min, 95% B. Samples were kept at 4 °C, analyzed randomly, and QC samples were included to ensure system stability and data accuracy.

Mass spectrometry was conducted on an Orbitrap Exploris™ 48, using ESI with conditions including 50 °C nebulizer gas, 50% Gas1, 2% Gas2, and temperatures/voltages for ionization. The primary range was 70–1200 Da with 60,000 resolution and 100 ms accumulation. Secondary acquisition followed similar parameters with a 4s dynamic exclusion.

### 2.4 Statistical analysis

Demographic and clinical data were presented as mean ± standard deviation (SD) for normally distributed variables or median (interquartile range, IQR) for non-normally distributed variables. Categorical variables were expressed as frequencies (percentages,%). Group comparisons were performed using Student’s t-test or Mann-Whitney U test for continuous variables, and Chi-square test for categorical variables. In order to see the diagnostic effect of different microbial groups, we made a receiver operating characteristic (ROC) curve analysis and calculated the area under the curve (AUC). All statistical analyses were performed using R software (v3.4.2). *P* < 0.05 was considered statistically significant.

## 3 Data analysis

### 3.1 Analysis of the microbiota

The number of valid tags varies from 56,112 to 125,609, with an average of 98,160. The dilution curve reached a stable point, and all samples met the standard of 55,000 reads, which indicated that the sequencing depth was sufficient. We used the UPARSE algorithm in Usearch (version v11.0.667), and divided clean tags into different operational taxonomic units (OTUs) according to 97% similarity (24). We also used the tool UCHIME (25) to remove those chimeric sequences. For OTU analysis, the most frequent sequence in each group was selected as the representative. We used RDP classifier (version v2.2) and SILVA database (version v138.2) for classification. In the R environment, we calculated various alpha diversity indices with standard formulas, including Sob, Chao1, ACE, Shannon and Simpson. In order to evaluate whether the data is enough, we draw a diversity dilution curve with ggplot2 package (version 3.4.2) in R. The differences of alpha diversity between groups were analyzed by Vegan package (version 2.6-4). The difference significance of alpha diversity index between the two groups was calculated by Welch’s t-test, and the default significance level was *P* < 0.05. Beta diversity analysis used OTU representative sequences. Unless otherwise specified, all Welch’s t-tests were double-tailed. Vegan package (version 2.6-4) also used to do PCoA analysis based on bray-curtis distance, and the outcomes were visualized with ggplot2 (version 3.4.2). In addition, PERMANOVA (also known as Adonis) also ran in Vegan package. We drew a bar chart to show the distribution of different species at the three taxonomic levels, especially the top ten most common species, and the rest were classified as “Other” or “Unclassified”. Using VennDiagram to draw Venn diagram, we can see which species are common to all groups and which are unique to some groups. In order to find out the biomarker species in each group, we used LEfSe software (version 1.0), and the selection criteria were that the linear discriminant analysis (LDA) score was at least 3, and *P* < 0.05. We also drew a box chart, and used the results of Welch’s t-test (*P* < 0.05) to show the quantitative changes of microbial species between different groups at these three classification levels. Through intersection analysis, we found out the core microbial groups with obvious differences at these levels. For the Random Forest (RF) model used to evaluate the diagnostic potential of key microbial communities, we employed 5-fold cross-validation (5-fold CV) to mitigate the risk of overfitting. Due to the small sample size and class imbalance, we did not perform nested feature selection or hyperparameter tuning, as the primary goal was exploratory feature evaluation rather than optimization of predictive accuracy. We used default RF parameters (ntree=500, mtry=sqrt(p)) and preselected features based on differential abundance analysis. Class weighting was not applied, which may bias performance toward the majority class. Confidence intervals for AUC were not calculated given the limited sample size. In this procedure, the dataset was randomly partitioned into 5 equal-sized folds. The model was trained on 4 folds and tested on the remaining 1 fold, iterating 5 times so that each fold served once as the test set. The reported AUC and accuracy represent the average performance across the 5 test folds, reflecting out-of-sample predictive ability rather than training accuracy. ROC curve analysis was performed based on the cross-validated predictions to calculate the AUC.

In order to predict metabolic pathways, we used the database of Kyoto encyclopedia of genes and genomes (KEGG) and PICRUSt2 software (version 2.5.3). We compared the functional differences at different levels of KEGG with the results of STAMP analysis and box diagram in PICRUSt2. If the two groups of box charts are separated more obviously, it means that the statistical difference between them may be greater. We used Welch’s t-test to test these differences. Then, we explored the relationship between the clinical characteristics and microbial community composition of these two groups of patients by drawing a heat map on the Omicsmart online analysis platform (http://www.omicsmart.com), and showed these relationships with Spearman’s rank correlation coefficient.

### 3.2 Analysis of the metabolomics

After the preparation of the sample, we identified the metabolites with AB SCIEX Triple TOF 6600 mass spectrometer. Two ionization methods, positive ion mode (POS) and negative ion mode (NEG), were used in the detection process. The data of each ionization method were analyzed separately. Orthogonal partial loss squares discriminant analysis (OPLS-DA) was completed with R package ropls (version 1.30.0). In order to further observe the changes of the abundance of differential metabolites, we also drew a volcano map. The x-axis represented the logarithmic multiple of the numerical change under the two conditions, and the y-axis represented the false discovery rate (FDR) or the negative logarithm of the *P* value. Different colors represented metabolites significantly increased or decreased according to the set standards, and blue marks indicated no significant changes. The set standards were: FC≥1, *P* ≤ 0.05, VIP ≥ 1.0.

Then we used OPLS-DA analysis to find out the important metabolites, and it used VIP score to measure the effectiveness of each metabolite in distinguishing different groups. After that, we standardized the found differential metabolites with z-score, and then made cluster analysis. Finally, we drew a heat map with pheatmap package to show their accumulation patterns. In order to further analyze the data, we did KEGG pathway enrichment analysis, and found out the pathways that were significantly richer than the whole metabolic group by hypergeometric test. Then the bubble charts of ClusterProfiler and Enrichplot were used to show the most important paths, and the top 10 KEGG paths ranked by *P* value were highlighted.

In order to combine the data of microbiome and metabolomics, we calculated Spearman’s correlation coefficients in R, and analyzed the relationship between intestinal microorganisms and metabolites at different classification levels. Spearman’s correlation can measure the degree of correlation between two variables and see how they change together, so that the relationship between microbial population and metabolic compounds can be evaluated.

## 4 Results

### 4.1 Demographic data and clinical characteristics of the subjects

A total of 69 children were enrolled in this study, including 53 people who were diagnosed with AD, and 16 healthy people of similar age and gender as the HC group. There was no significant difference in average age (6.47 ± 2.76 vs. 6.12 ± 2.28 years, *P* = 0.615) or in gender distribution (54.7% vs. 56.2% male, *P* = 1.000) between AD group and HC group. The AD group exhibited mild to moderate disease severity, with a median EASI score of 5.95 (IQR: 4.00-9.20) and a median IGA score of 2.00 (IQR: 2.00-3.00). The mean POEM, DFI, and CDLQI scores were 11.62 ± 5.76, 11.49 ± 6.23, and 10.03 ± 5.29, respectively (**Table 1**).

**Table 1:** Demographic data and clinical characteristics of AD patients and HC.

Characteristics	AD (*N* = 53)	HC (*N* = 16)	*P* value
Age	6.47 ± 2.76	6.12 ± 2.28	0.615
Gender			1.000
Male	29 (54.7%)	9 (56.2%)	
Female	24 (45.3%)	7 (43.8%)	
EASI	5.95 (4.00, 9.20)	/	/
IGA	2.00 (2.00, 3.00)	/	/
POEM	11.62 ± 5.76	/	/
DFI	11.49 ± 6.23	/	/
CDLQI	10.03 ± 5.29	/	/

Legend: AD, atopic dermatitis; HC, healthy control; EASI, Eczema Area and Severity Index; IGA, Investigator’s Global Assessment; POEM, Patient-Oriented Eczema Measure; DFI, Dermatitis Family Impact; CDLQI, Children’s Dermatology Life Quality Index; *P* < 0.05 was considered significant.

### 4.2 Gut microbiota analysis

#### 4.2.1 Gut microbiota community characteristics

**Figure 1:**
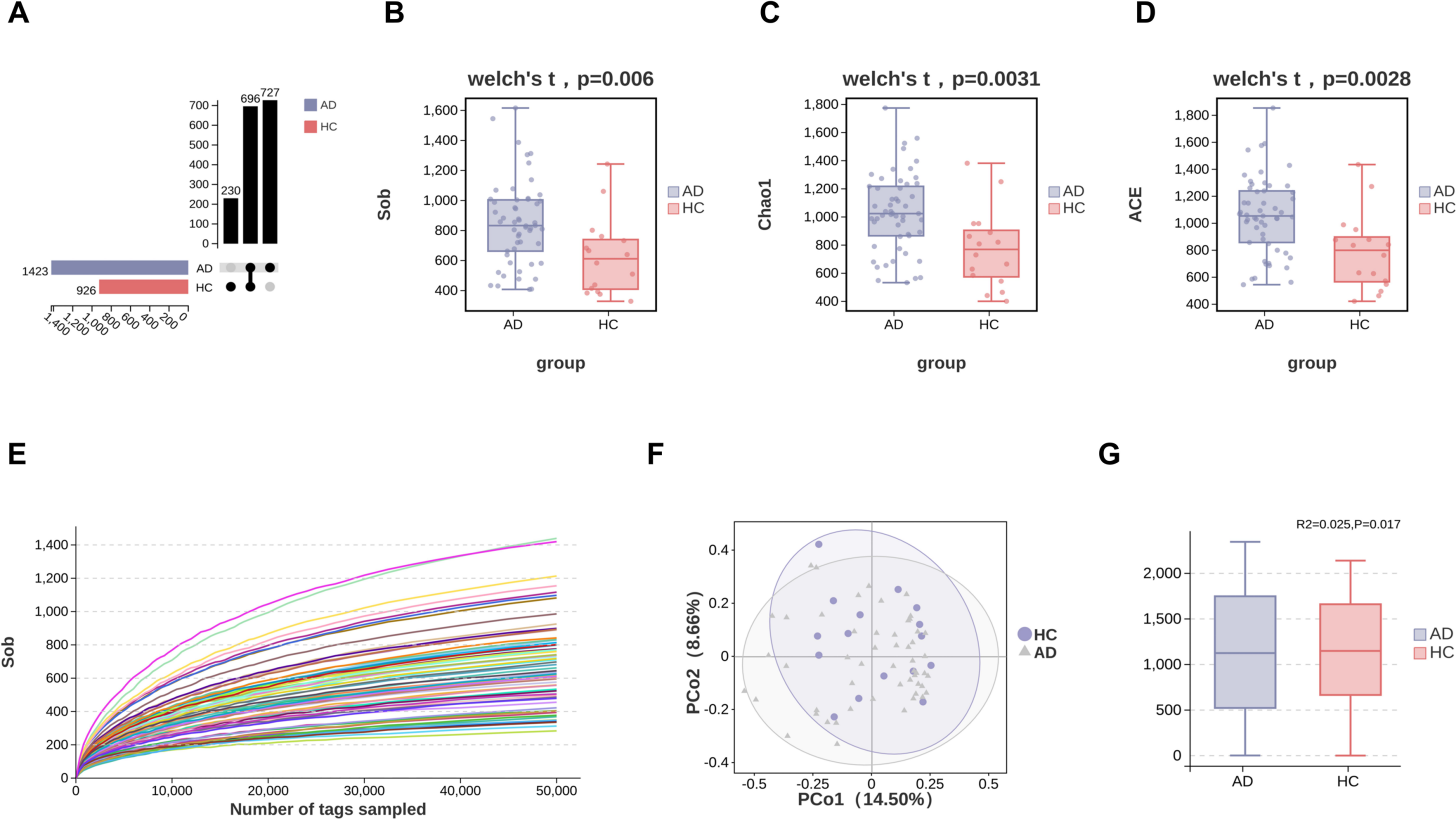
**(A)** Upset plot showing the number of OTUs per group. Significant difference in a diversity between AD and HC populations, as estimated by **(B)** Sob index, **(C)** Chao 1 index and **(D)** ACE index. **(E)** Sparsity curve analysis. **(F)** β-diversity analysis based on PCoA plot. **(G)** PERMANOVA analysis based on bray-curtis distances.

We sequenced the fecal samples of all the participants in the study. A total of 2,349 distinct OTUs were identified across all samples, including 1,423 OTUs from AD group and 926 OTUs from HC group. There are 696 OTUs that are the same between the two groups, but there are 727 unique OTUs in AD group and 230 unique OTUs in HC group (**Figure 1A**). This shows that people diagnosed with AD have more kinds of intestinal flora than healthy people. α-diversity is used to describe the degree of difference within intestinal microbial communities, mainly depending on species richness and uniformity. α-diversity analysis shows that the Sob, Chao1 and ACE values of AD group are significantly higher (**Figures 1B–D**), but Simpson and Shannon index of AD group are only slightly higher, with little difference (**Supplementary Figures 1A, B**).

This indicated that the intestinal microbial community was more abundant in the AD group than in the HC group, while the diversity was not significantly different between groups. In order to reduce the possible bias caused by different sequencing depths, all samples were uniformly adjusted to 55,000 Rarefaction Depth per sample, and then the diversity was analyzed. Sparse curve analysis shows that the sample size is sufficient, because the sequencing reading has tended to be stable (**Figure 1E**). In addition, PCoA analysis of β-diversity based on OTU’s Bray-Curtis distance showed that the microbial community structure of the two groups partially overlapped but could still be distinguished (Permanova: R^2^ = 0.025, *P* = 0.017) (**Figures 1F, G**). The results show that there are differences in microbial community makeup between the two groups of samples.

#### 4.2.2 Microbial community identification and species distribution

**Figure 2:**
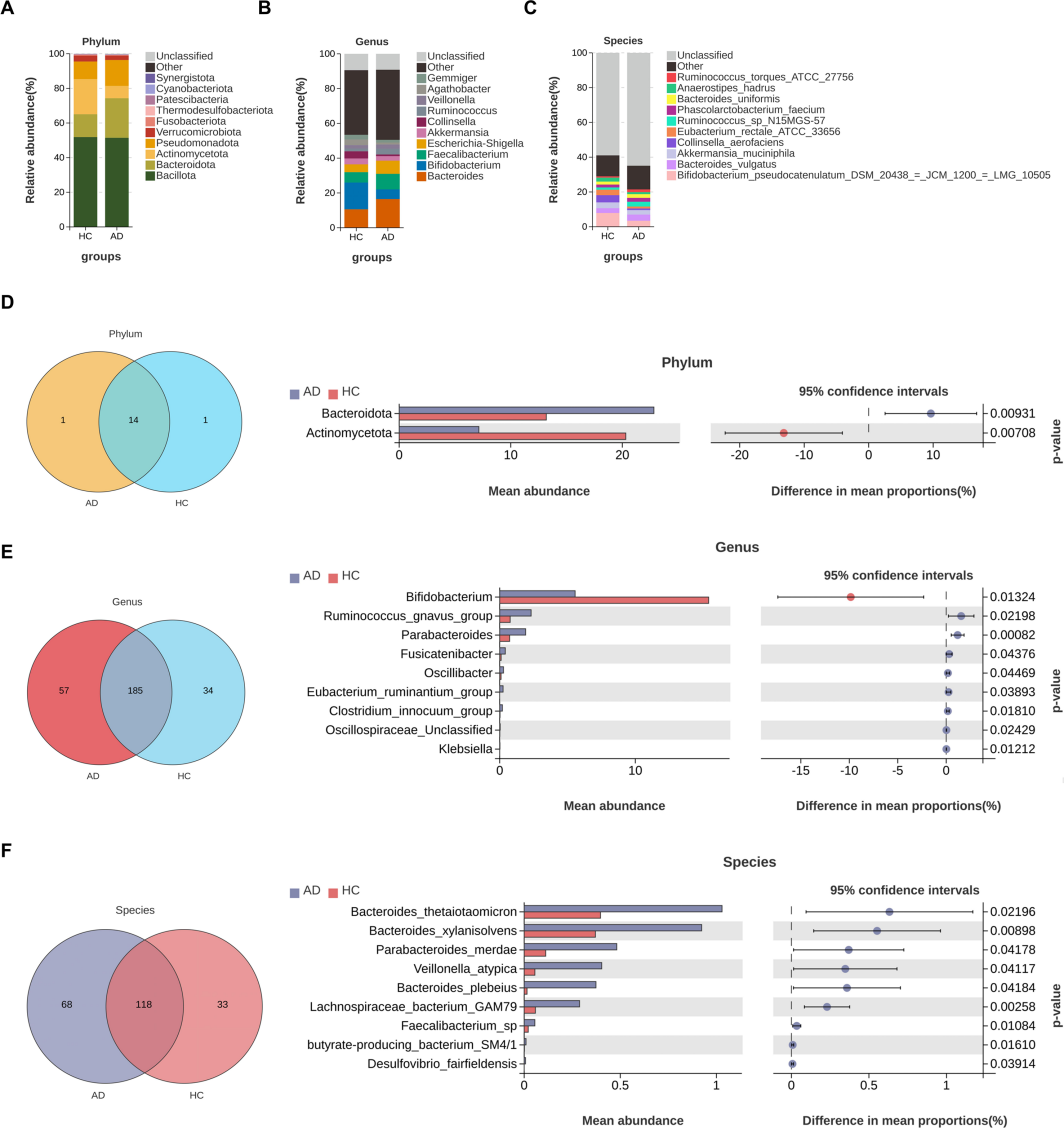
Subgroup analysis of gut microbiota composition at phylum **(A)**, genus **(B)**, and species **(C)** levels. Statistically significant bacterial composition in the AD and HC groups at phylum **(D)**, genus **(E)**, and species **(F)** levels.

In the AD and HC groups, the five most important phyla were Bacillota, Bacteroidota, Actinomycetota, Pseudomonadota and Verrucomicrobiota (**Figure 2A**). Bacillota accounted for the highest proportion of 51.44% and 51.85% in AD and HC, respectively. And *Bacteroides*, *Bifidobacterium*, *Faecalibacterium*, *Escherichia-Shigella*, *Akkermansia*, *Collinsella*, *Ruminococcus*, *Veillonella*, *Agathobacter* and *Gemmiger* were the top 10 genera at the genus level (**Figure 2B**). *Bifidobacterium_pseudocatenulatum_DSM_20438_=_JCM_1200_=_LMG_10505*, *collinsella_aerofaciens*, *akkermansia_muciniphila*, *eubacterium_rectale_ATCC_33656*, *bacteroides_vulgatus*, *anaerostipes_hadrus*, *bacteroides_uniformis*, *phascolarctobacterium_faecium*, *ruminococcus_sp_N15MGS-57 and ruminococcus_torques_ATCC_27756* were the top 10 OTUs classified to species-level taxa (**Figure 2C**). We caution that 16S rRNA V3-V4 amplicon sequencing with 97% OTU clustering does not reliably resolve true species. These assignments are tentative and should be validated by shotgun metagenomic sequencing.

At the phylum level, it was found that compared with the HC group, the Bacteroidota in AD group increased significantly, but the Actinomycetota decreased significantly (**Figure 2D**). At the genus level, *Ruminococcus_gnavus_group*, *Parabacteroides*, *Fusicatenibacter*, *Oscillibacter*, *Eubacterium_ruminantium_group*, *Clostridium_innocuum_group*, *Oscillospiraceae_Unclassified* and *Klebsiella* increased and *Bifidobacterium* decreased in AD groups (**Figure 2E**). *Bacteroides_thetaiotaomicron*, *bacteroides_xylanisolvens*, *parabacteroides_merdae*, *veillonella_atypica*, *bacteroides_plebeius*, *lachnospiraceae_bacterium_GAM79*, *faecalibacterium_sp*, *butyrate-producing_bacterium_SM4/1* and *desulfovibrio_fairfieldensis* were more abundant in AD patients at the putative species level (OTU-based classification) (**Figure 2F**).

**Figure 3:**
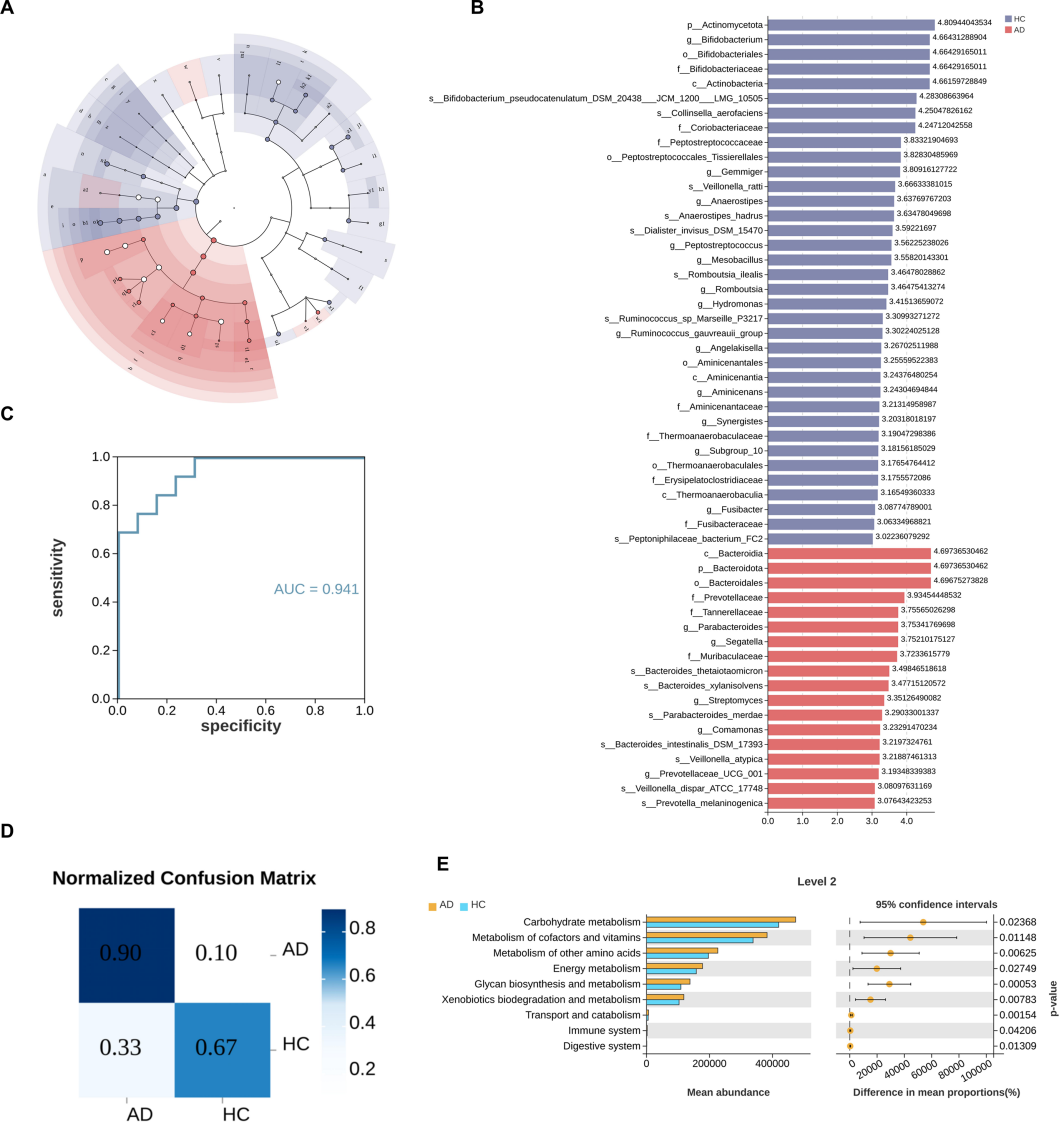
LEfSe used to identify essential differences in bacterial abundance (Domain to species level) between the AD and HC groups. Dendrogram **(A)** and bar graph **(B)** are used to show Only taxa with a significant LDA score ≥ 3. ROC curve **(C)** and confusion matrix **(D)** showed the combined ability of Bacteroidota*, Parabacteroides, bacteroides_plebeius, bacteroides_xylanisolvens, bacteroides_thetaiotaomicron and parabacteroides_merdae* biomarkers in predicting AD. **(E)** Functional annotations at levels 2.

In addition, we also used the method of Lefse to find out the most important bacterial species in AD group and HC group. It was found that compared with HC group, there were more bacteria such as Bacteroidota, Bacteroidia and Bacteroidales in AD group, and the most bacteria in HC group was Actinomycetota (**Figures 3A, B**). The key differential microbes at the intersection of phylum, genus, and putative species levels were Bacteroidota, *Parabacteroides*, *bacteroides_plebeius*, *bacteroides_thetaiotaomicron*, *bacteroides_xylanisolvens* and *parabacteroides_merdae*. By applying RF regression on relative abundance data from these core microbes using 5-fold cross-validation, the average cross-validated AUC for Bacteroidota, *Parabacteroides*, *bacteroides_plebeius*, *bacteroides_thetaiotaomicron*, *bacteroides_xylanisolvens* and *parabacteroides_merdae* were 0.686, 0.750, 0.601, 0.680, 0.665 and 0.748, respectively (**Supplementary Figures 1C–H**). These could effectively differentiate AD from HC, but their diagnostic values were quite limited. When these phyla, genera and species were used in combination for diagnosis, the cross-validated combined AUC was 0.941, with an accuracy of 84.6% (**Figure 3C**). The confusion matrix showed that, under the current diagnostic threshold, the sensitivity of model for identifying the AD group was 90.0% (**Figure 3D**). This indicated a relatively good combined diagnostic outcome.

#### 4.2.3 Functional analysis of microbiome genes and relationship between clinical characteristics

In addition, we also used PICRUSt2 to check the 16s rRNA sequence data, paying special attention to the classification information of the second and third levels. The five main biochemical pathways in AD group and HC group include carbohydrate treatment, amino acid decomposition, coenzyme and vitamin management, terpenoids and polyketides treatment, and modification of other amino acids (**Supplementary Figures 2A, B**). Regarding the functional differences between the two groups of microbial communities, we found that five secondary functions changed most obviously. These findings show that the microbial composition in patients is obviously related to carbohydrate metabolism, metabolism of cofactors and vitamins, metabolism of other amino acids, energy metabolism and glycan biosynthesis and metabolism (**Figure 3E**). At the third level, we evaluated the five most significant enrichment difference functions. Compared with HC group, the intestinal microflora of AD group is related to biotin metabolism, carbon fixation pathways in prokaryotes, folate biosynthesis, citrate cycle (TCA cycle) and pentose and glucuronate interconversions (**Supplementary Figure 2C**). These predicted differences suggest potential enrichment of the pathways in AD-associated microbiomes, but these findings require validation by metatranscriptomics or targeted metabolomics.

**Figure 4:**
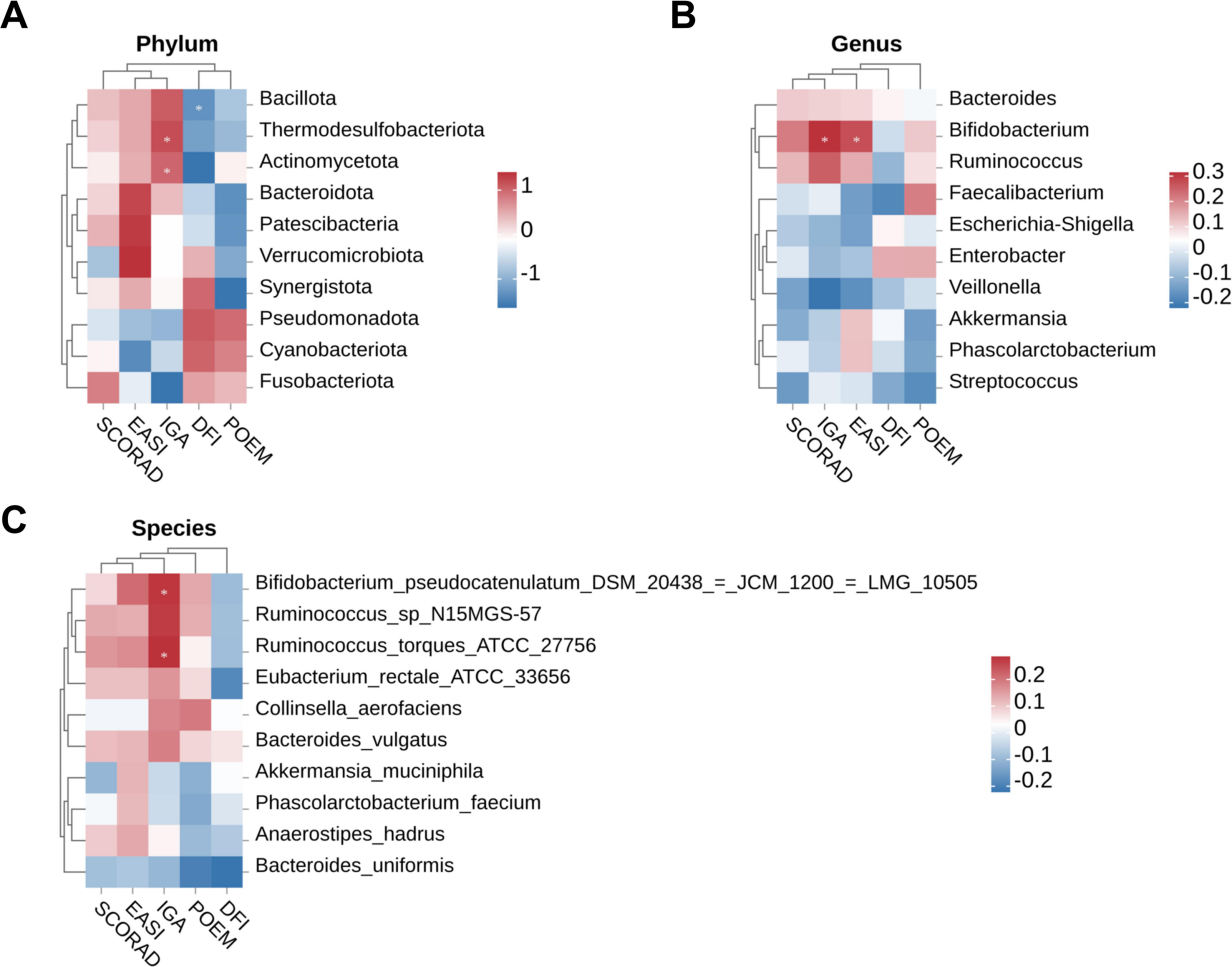
Spearman correlation heatmap between microbiota and clinical indicators at phylum **(A)**, genus **(B)**, and species **(C)** levels. ^*^P < 0.05, ^**^P < 0.01, ^***^P < 0.001.

Moreover, in Spearman correlation analysis, IGA exhibited positive correlations with multiple gut microbiota, including Thermodesulfobacteriota, Actinomycetota, *Bifidobacterium*, *bifidobacterium_pseudocatenulatum_DSM_20438_=_JCM_1200_=_LMG_10505* and *ruminococcus_sp_N15MGS-57*. *Bifidobacterium* also showed a positive correlation with EASI. Bacillota demonstrated a negative correlation with DFI (**Figures 4A–C**).

### 4.3 Differences in metabolites between two patient groups

**Figure 5:**
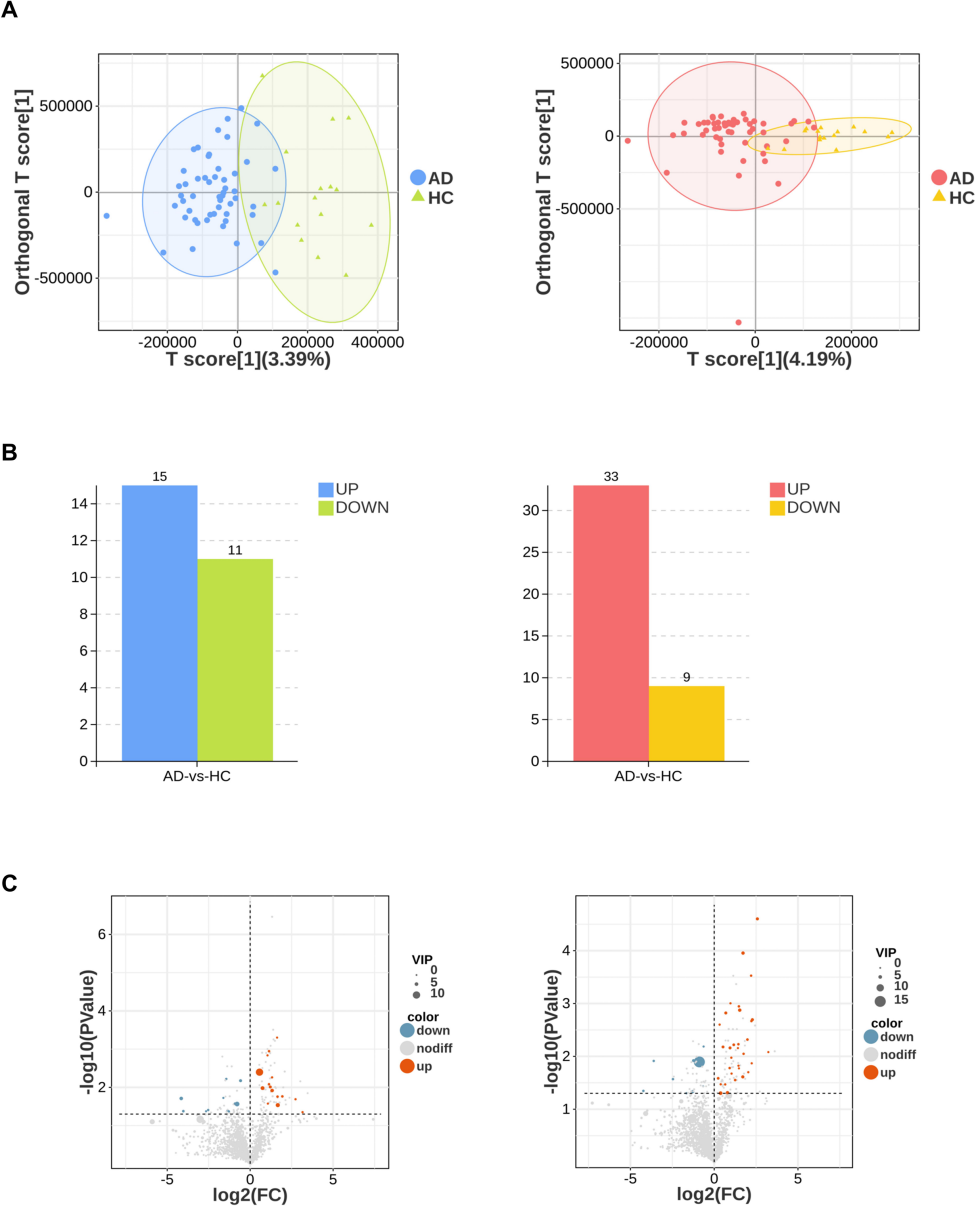
**(A)** OPLS-DA scoring plots in NEG and POS modes. **(B)** Bar charts of the number of up-regulated and down-regulated differential metabolites in NEG and POS modes. **(C)** Volcano plot: x-axis, log2FC; y-axis, -log10 (*P*-value); red/blue indicate up/down-regulated metabolites; dot size represents VIP.

Then, we investigated the non-targeted metabolites in fecal samples obtained from AD patients and HC. Through the OPLS-DA model, we found that the metabolic characteristics of the two groups are obviously different, and they exhibited distinct characteristics in both positive and negative ion modes (**Figure 5A**).

**Figure 6:**
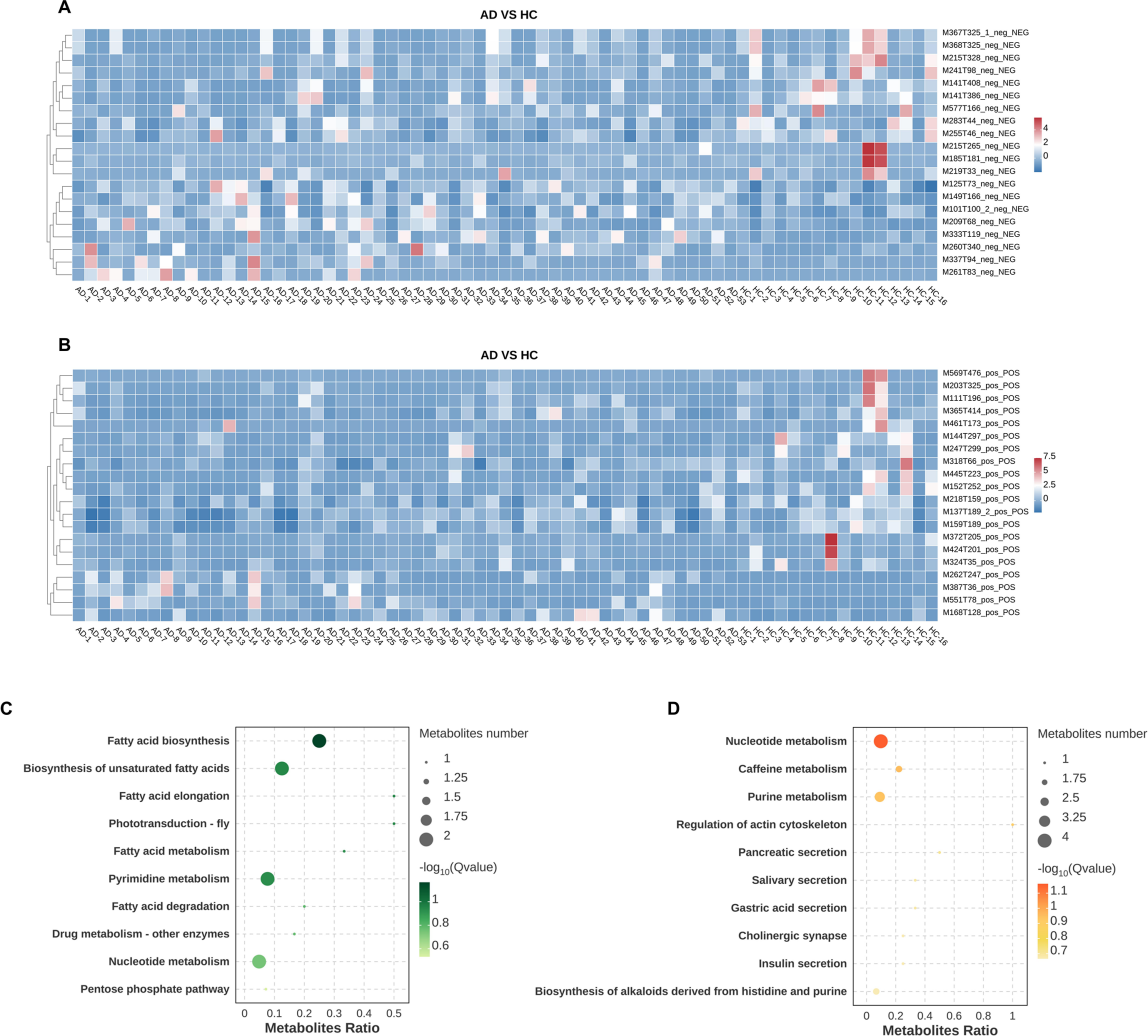
Differential metabolites and KEGG pathways. **(A, B)** Heatmaps in NEG and POS modes. The colors from blue to red indicate the relative contents of the metabolites in the two groups. **(C, D)** Top 10 KEGG pathways in NEG and POS modes.

In patients with AD and HC, 1,978 metabolites with positive ion mode and 1,591 metabolites with negative ion mode were detected. Compared with HC, 15 kinds of metabolites were upregulated, while 11 kinds of metabolites were downregulated for AD in negative ion mode. 33 kinds of metabolites were upregulated, while 9 kinds of metabolites were downregulated for AD in positive ion mode (**Figures 5B, C**). We did cluster analysis on the metabolites with different expression levels, and showed the unique metabolic profiles of each cluster intuitively with heatmaps (**Figures 6A, B**). According to the standards of VIP >1, FC ≥1 or FC ≤1, *P* < 0.05, we found 68 metabolites with different accumulation (**Supplementary Table 1**). Therefore, we chose these metabolites as the benchmark for further research. In addition, through KEGG annotation for metabolic enrichment and pathway analysis, we found that these metabolites with great changes are related to their corresponding biochemical pathways. They are mainly involved in lipid metabolism, including fatty acid biosynthesis, biosynthesis of unsaturated fatty acids, fatty acid elongation and fatty acid degradation in negative ion mode. In the positive ion mode, we found that the metabolites with different expressions are also closely associated with the metabolic pathways such as nucleotide metabolism, caffeine metabolism and purine metabolism in positive ion mode (**Figures 6C, D**).

### 4.4 Cross-correlation analysis between the microbiota and metabolites

**Figure 7:**
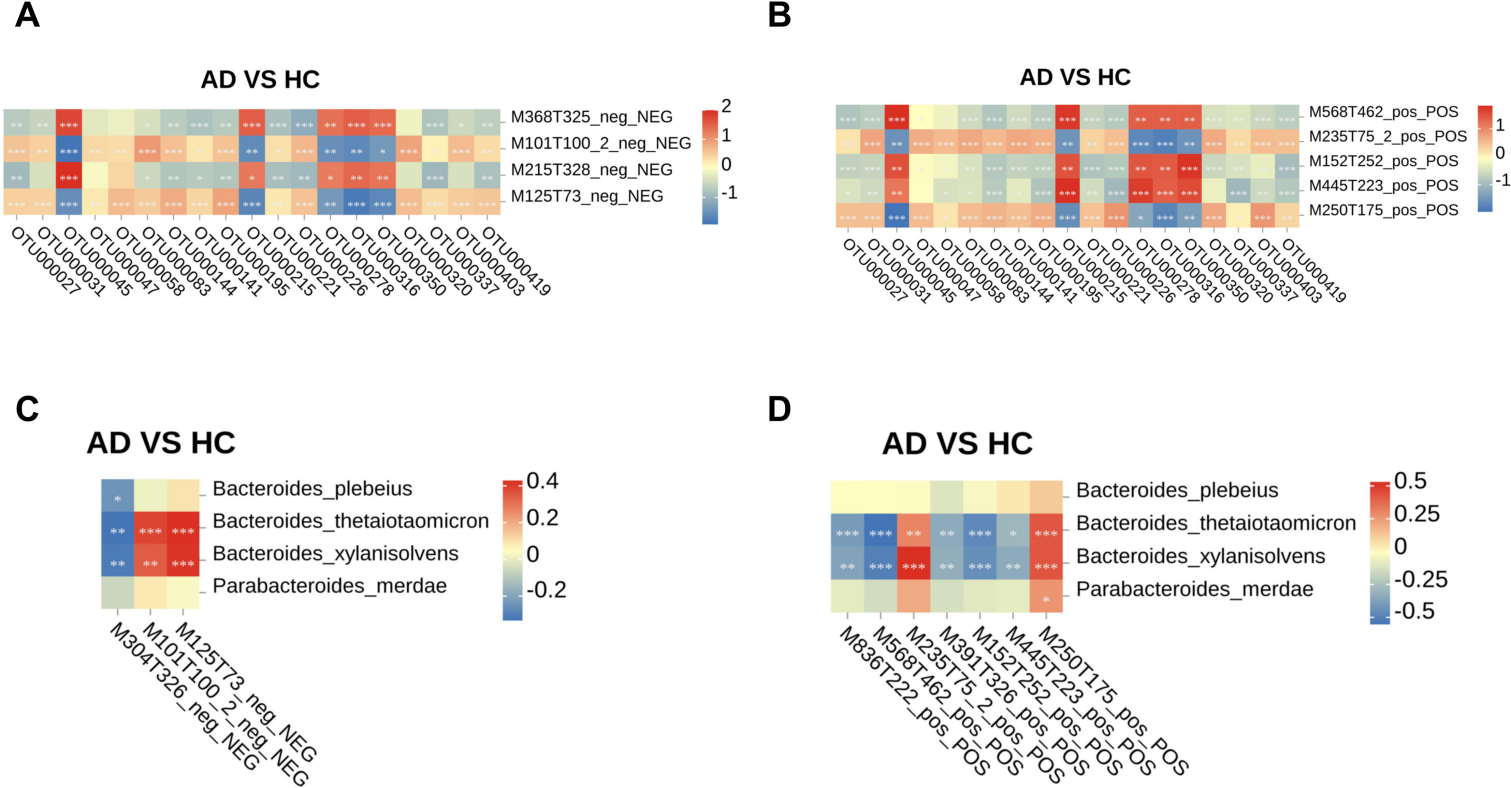
Microbiota-metabolite correlations. **(A, B)** Spearman heatmaps of top 30 OTUs and metabolites. **(C, D)** Correlation heatmaps of four key species and metabolites in NEG and POS modes. Red: positive correlation; blue: negative correlation. ^*^P < 0.05, ^**^P < 0.01, ^***^P < 0.001.

In order to explore the relationship between the differences of intestinal flora and fecal metabolites in AD patients compared with HC, we used Spearman correlation coefficient to do correlation analysis. The analysis covers microorganisms and metabolites found at the OTU level, and there is a statistically significant correlation between them. We drew a heat map to visually show the strength and direction of these relationships. We selected 30 OTUs with the strongest correlation and the metabolites accumulated by differences, and then displayed them according to the correlation coefficient. Then the correlation heat map is used to draw the correlation between intestinal flora and metabolites (**Figures 7A, B**). This shows that there is a strong relationship between intestinal metabolites and the microbiome structure of children with AD. Then, we made a cross-correlation analyses on four main microbial groups with strong diagnostic potential (*bacteroides_plebeius*, *bacteroides_thetaiotaomicron*, *bacteroides_xylanisolvens* and *parabacteroides_merdae*) and metabolites with obvious changes in abundance (**Figures 7C, D**).

## 5 Discussion

In this study, we examined the gut microbiota and metabolic profiles in children with mild-to-moderate AD versus HC. We hope to identify some potential biomarkers for non-invasive diagnosis and support strategies that focus on preventing and managing early-onset AD. Alterations in the gut microbiota and its derived metabolites hold promise as non-invasive biomarkers for aiding in the auxiliary diagnosis of AD. Fecal sampling for microbiota or metabolite analysis provides a simple, painless, and repeatable method that avoids the invasiveness of blood draws or skin biopsies. This non-invasive approach is particularly advantageous in pediatric populations. In this study, significant variations in gut microbiome composition and distribution were observed between the groups, with AD patients showing unique metabolic patterns linked to their gut microbiota.

In the component of gut microbiota, we found a significant increase in the species richness index for AD, while the evenness index, although slightly elevated, did not reach statistical significance. This finding indicates that the number of species colonizing the gut of AD has significantly increased, but the relative abundance proportions of individual species have not changed significantly and remain largely similar to those of healthy individuals. Typically, an increase in microbial richness could imply the colonization or proliferation of more opportunistic bacterial communities within the gut environment, which is relevant to the potential slowing of gut motility, compromised gut barrier function, or chronic inflammatory states observed in AD. This provides a niche for these non-resident microbial communities. However, the evenness index did not show a significant change, suggesting that despite the increased acceptance of more species by AD, the core framework of the entire microbial community, comprising the dominant proportion of core bacteria that maintain basic gut functions, remains relatively stable. β-diversity analysis showed that the composition of microbial communities in different groups was quite different (Permanova: R^2^ = 0.025, *P* = 0.017). The results show that the main factor affecting the development of AD may be the specific composition of microbial community, not the total number of species.

The study found that the intestinal microbial composition of the two groups was different. At the phylum level, the phylum Bacteroidota was significantly elevated in AD patients, while the phylum Actinomycetota was significantly reduced (**Figure 2D**). Multiple studies have shown that the abundance of Bacteroidota and Actinomycetota in the skin microbiome of AD patients undergoes significant changes, and that the extent of these changes correlates with the severity of the disease (26, 27). In AD infant feces, there is a significant enrichment of *Bacteroidaceae* (a genus within *Bacteroidota*). This genus may contribute to the inflammatory response by promoting the secretion of IL-17 by Th17 cells. Actinomycetota is a beneficial microbial community for healthy skin. The cell wall component lipoteichoic acid (LTA) can permeate into the dermis and influence the function of mast cells through Toll-like receptors. In sterile animal models, the absence of such microorganisms has been shown to lead to abnormal development of the immune system, providing indirect support for their role in maintaining skin immune homeostasis (28). A decrease in the abundance of Actinomycetota in AD patients is associated with compromised barrier function (29). At the genus level, the abundance of *Parabacteroides* and *Klebsiella* was increased in AD group, whereas that of *Bifidobacterium* was decreased (**Figure 2E**). At the species level, *parabacteroides_merdae*, *bacteroides_thetaiotaomicron*, *bacteroides_xylanisolvens*, *bacteroides_ plebeius* were more abundant in AD patients (**Figure 2F**). Several studies have found that the relative abundance of *Parabacteroides* in the intestinal tract of AD patients is significantly higher than that of HC. For example, 16S rRNA sequencing revealed elevated abundance of this genus in the gut microbiota of adult AD patients (30). In 6-month-old infants, its abundance was positively correlated with IgE level and SCORAD score, and AD children with rs2275913 variant had higher levels (31). The abundance of *Parabacteroides* and *Bacteroides* was higher in the severe AD patients than in the mild AD patients, and the SCORAD score was positively correlated with *Parabacteroides* and *Bacteroides (*32). Another study reported an increased abundance of *Parabacteroides* and a decreased abundance of *Bifidobacteria* in the intestinal tract of children with AD (33). A previous prospective study also found that decreased levels of early *Bifidobacterium* colonization were related to an increased risk of AD (34). In addition, *Klebsiella* related functional pathways were significantly enriched in the skin, oral cavity and gut microbiota of AD children, suggesting that *Klebsiella* may play a role in the development of AD (27). The alpha-type phenol-soluble modulin (PSM) toxin secreted by *Klebsiella* can significantly induce the gene expression and release of pro-inflammatory factors such as CXCL8, CCL20, TNF-α, and IL-6 in human keratinocytes; it also triggers a strong inflammatory response in an ex vivo human skin tissue model, suggesting that it participates in the onset of AD by exacerbating local cutaneous inflammation (35). Further analysis by RFmethod showed that microorganisms such as Bacteroidota, *Parabacteroides*, *bacteroides_plebeius*, *bacteroides_xylanisolvens*, *bacteroides_thetaiotaomicron* and *parabacteroides_merdae* may be important biomarkers. These demonstrated high combined predictive accuracy for mild-to-moderate pediatric AD, with AUC = 0.941.

We analyzed the functional changes with PICRUSt2, and found that the microflora in mild-to-moderate pediatric AD patients changed obviously in many metabolic processes. Especially related to carbohydrate metabolism (e.g., glyoxylate and dicarboxylate metabolism, citrate cycle and pentose and glucuronate interconversions) and metabolism of cofactors and vitamins (e.g., lipoic acid metabolism, folate biosynthesis and biotin metabolism). These changes in biological pathways may affect the formation of SCFAS, the regulation of immune response and the integrity of skin barrier, thus playing a role in the development of AD. For instance, A study on children with early AD found that the intestinal microbiome of children with AD has significant dysfunction in carbohydrate metabolism, cofactor and vitamin metabolism pathways, and these changes are related to the host immune response (36). However, these findings are based on computational inference from 16S data and should not be interpreted as direct evidence of functional activity. Future studies employing shotgun metagenomics or metatranscriptomics are needed to confirm these functional predictions. Spearman correlation analysis found that there was a correlation between the changes of intestinal microbial community and the clinical indexes of AD. The disease severity indexes of mild-to-moderate pediatric AD patients were positively correlated with Thermodesulfobacteriota, Actinomycetota and *Bifidobacterium*. The quality of life score was negatively correlated with Bacillota. Diisocyanates, an environmental pollutant, have been found to disrupt the ability of beneficial bacteria such as Thermodesulfobacteriota to produce protective glycerides and ceramide, leading to the imbalance of the skin microbiome, suggesting that the impaired function of Thermodesulfobacteriota may be involved in the pathogenesis of AD (37). Furthermore, multiple studies have shown that the relative abundance of Actinomycetota in the skin microbiome of children with AD significantly increases, and this increase is directly related to the severity of the disease. Additionally, when the condition improves following treatment, the abundance of Actinomycetota decreases (38). In infants with AD, an increase in the abundance of Actinomycetotas in the gut is positively correlated with IgE levels and SCORAD scores, which is consistent with the findings of this study (31). Nevertheless, in contradiction to previous studies, which supported the protective role of *Bifidobacterium* in AD, the positive association between *Bifidobacterium* and the severity of AD was observed in this study (12, 34, 39). Existing evidence suggested that Bacillota plays a dual role in AD: changes in its overall abundance may serve as a disease risk marker, while loss or abnormal metabolic function of specific functional strains within the phylum, such as butyrogenic bacteria, may be involved in pathological processes (31, 40).

Through non-targeted metabonomics, our research has found special metabolic characteristics, which can distinguish people with AD from healthy people. Among AD patients, 48 metabolites were significantly enriched, while only 20 decreased, indicating that their overall metabolic function has been generally enhanced. OPLS-DA model shows that there is obvious difference between the two groups. KEGG pathway enrichment analysis showed that these metabolites were mainly related to lipid metabolism, including fatty acid biosynthesis, biosynthesis of unsaturated fatty acids, fatty acid elongation and fatty acid degradation in negative ion mode. This was consistent with the previous research results (41). Current evidence suggests that the lipid metabolic disorders associated with AD are a complex, multi-level, and multi-pathway pathological process: at the local skin level, it manifests as abnormalities in ceramide chain length and impaired fatty acid elongation (42, 43); at the systemic level, it involves imbalances in polyunsaturated fatty acid levels and immune activation mediated by specific plasma lipids (44, 45); microbial interactions further exacerbate metabolic abnormalities (27).

The significant change of metabolite level is also related to the pathway involved in nucleosynthesis, caffeine metabolism and purine metabolism in positive ion mode. At present, a number of metabolomics studies have clearly stated that no such association has been detected, but some studies have found that extracellular adenosine triphosphate (ATP, a purine nucleotide) can reduce the expression of filagalin and aggravate inflammation, and targeted inhibition of ATP release can alleviate the symptoms of AD (41, 46, 47). In addition, caffeine is widely used in dermatological products, but there is currently no direct evidence of an association between caffeine metabolism and AD (48).

In our study, Spearman correlation analysis shows that there may be a connection between intestinal microbiota and fecal metabolites. Notably, we observed that *bacteroides_thetaiotaomicron* and *bacteroides_xylanisolvens* abundances correlated with elevated levels of isovaleric acid. Current evidence suggests that isovaleric acid, as a metabolic product of intestinal microorganisms, may play a role in the development of AD by disrupting the serotonin synthesis pathway, and that its levels are associated with the clinical severity of the condition (49, 50). The results show that the irregular metabolic activity during the development of AD may be closely related to the function of gut microbiota. This further shows that the gut-skin axis plays an important role in managing mild-to-moderate pediatric AD. In the future, we may be able to detect AD earlier by detecting fmicrobiota and metabolites markers.

There are several limitations to this study. First, as a single-center cross-sectional study, we cannot establish causal or temporal relationships between gut microbiota dysbiosis, metabolic alterations, and the onset or progression of mild-to-moderate pediatric AD. Longitudinal studies with serial sampling are needed to track microbiome-metabolite dynamics, validate their predictive value for disease development, and establish directionality and causality. Second, a formal *a priori* power analysis was not performed, as this was designed as an exploratory single-center study. Sample size was primarily determined by the number of consecutive eligible participants recruited during the study period. We conducted a *post-hoc* power analysis using the observed effect size from the primary outcome. The results indicate that the current sample size provides sufficient statistical power for detecting the main group differences in microbial community structure. However, we acknowledge the limitations in detecting subtle differences among rare taxa due to the modest sample size and small effect size. Larger, multi-center prospective studies are required for further validation. Third, the healthy control group is relatively small compared with the AD group, resulting in an imbalanced design. Although *post-hoc* power calculations confirmed adequate power for the observed effect sizes, the modest HC sample size may limit detection of rare taxa/metabolites and reduce generalizability. Fourth, while the Random Forest model employed 5-fold cross-validation to mitigate overfitting, the overall sample size remains modest for high-dimensional microbiome data. Independent external validation cohorts will be essential. Fifth, although we controlled for recent antibiotic/probiotic use and major exclusion criteria, unmeasured confounders such as detailed dietary patterns, breastfeeding history, and environmental exposures could influence results. Functional validation of the observed microbiota-metabolite correlations through *in vitro* or animal models is warranted to establish mechanistic causality. Sixth, the use of 16S rRNA V3-V4 amplicon sequencing with 97% OTU clustering limits taxonomic resolution. We cannot reliably distinguish true species or strains; all species-level assignments are tentative and should be validated by shotgun metagenomic sequencing in future studies. Seventh, PICRUSt2-based functional predictions are computational inferences, not direct measurements of microbial gene expression or metabolic activity; they should be considered hypothesis-generating only. Eighth, our metabolomics statistical thresholds are exploratory; stricter thresholds and independent validation are needed to confirm potential metabolic biomarkers.

Several important avenues should be pursued by future studies to build upon our findings. First, large-scale, multicenter prospective cohort studies with balanced group sizes are urgently needed to validate the generalizability of our observed microbiome-metabolome associations and the diagnostic potential of our biomarker panel. Second, longitudinal studies tracking gut microbiota and metabolome dynamics from infancy through AD onset, progression, and treatment are essential to establish temporal sequences and identify early-life intervention windows. Third, external validation of our Random Forest-derived biomarker panel in independent pediatric populations from diverse geographic and ethnic backgrounds is required before any clinical application can be considered. Fourth, mechanistic studies using germ-free animal models, fecal microbiota transplantation, or *in vitro* systems are necessary to definitively establish causality between specific microbial taxa and AD pathogenesis. Fifth, interventional trials, including probiotics targeting Bifidobacterium, prebiotics, or metabolite supplementation, should be designed to test whether restoring gut microbial balance can ameliorate AD symptoms in children. Finally, integrating multi-omics data using systems biology approaches may enable personalized medicine strategies for pediatric AD management.

## 6 Conclusion

This study combined 16S rRNA sequencing with non-targeted metabonomics, and found that the changes of the structure of the gut microbiome and metabolic functions in children with mild-to- moderate AD were significant. Pediatric AD patients exhibited pronounced gut microbiome dysregulation and extensive metabolic disturbances. We also found that there is a strong relationship between the gut microbiome and metabolites, suggesting a potential interplay that warrants further mechanistic investigation. Using 5-fold cross-validated RF modeling, a combined panel of Bacteroidota, *Parabacteroides*, *bacteroides_plebeius*, *bacteroides_thetaiotaomicron*, *bacteroides_xylanisolvens*, and *parabacteroides_merdae* demonstrated promising exploratory predictive potential for distinguishing mild-to-moderate pediatric AD from healthy controls. This study provides new theoretical and experimental evidence regarding the gut-skin axis in children with mild to moderate AD. However, our findings are associative, require validation in larger independent cohorts, and should not yet be interpreted as establishing diagnostic biomarkers for clinical use. The results offer a foundation for future non-invasive diagnostic approaches and therapeutic strategies targeting the microbiome and metabolome, including the use of probiotics and metabolite-based supplements.

## Data Availability

The datasets presented in this study can be found in online repositories. China National Center for Bioinformation (GSA: CRA071715) that are publicly accessible at https://ngdc.cncb.ac.cn/gsa and OMIX (https://ngdc.cncb.ac.cn/omix: OMIX017284).
