# Supplementary material for: Non-invasive detection of pediatric atopic dermatitis based on fecal microbiota and metabolite profiles: a diagnostic approach
Source: Front Immunol. 2026 Jun 5;17:1836716. doi: 10.3389/fimmu.2026.1836716 (PMC13278929; doi:10.3389/fimmu.2026.1836716)
Supplement: Supplementary file 1 [file DataSheet1.pdf]

Supplementary Material

1 Supplementary Figures and Tables

1.1 Supplementary Figures

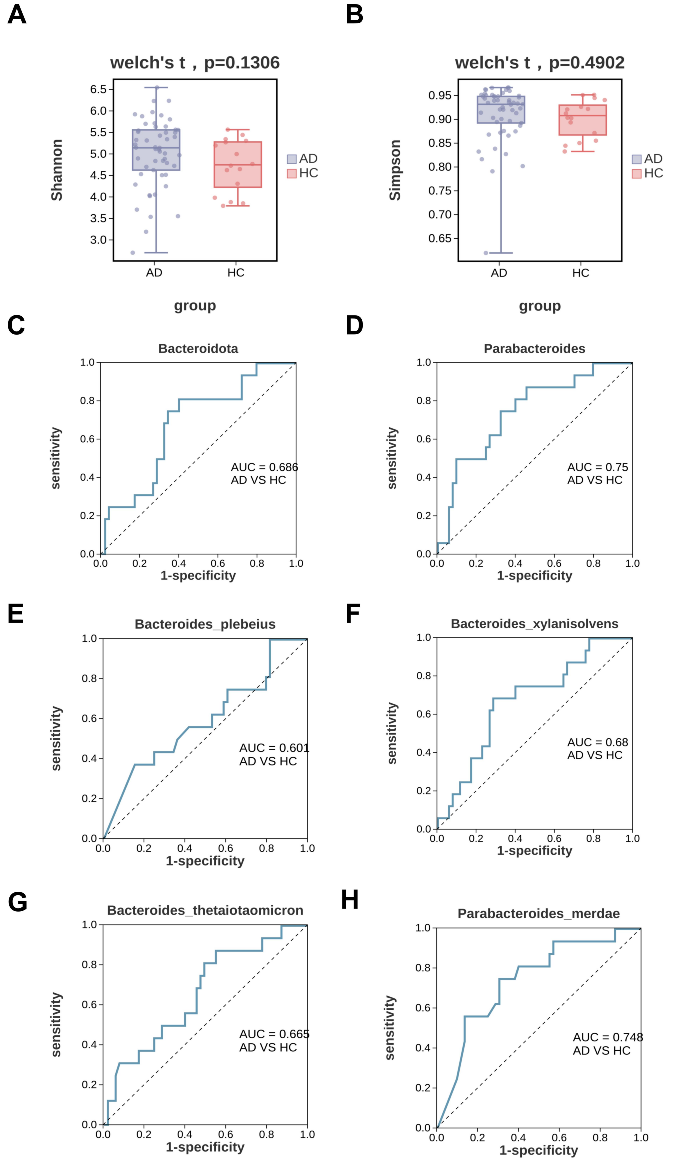

Supplementary Figure 1. No significant difference in a evenness between AD and HC populations, as estimated by (A) Shannon index, (B) Simpson index. ROC curves showed the ability of Bacteroidota (C), Parabacteroides (D), bacteroides_plebeius (E), bacteroides_xylanisolvens (F), bacteroides_thetaiotaomicron (G) and parabacteroides_merdae (H) biomarkers in predicting AD.

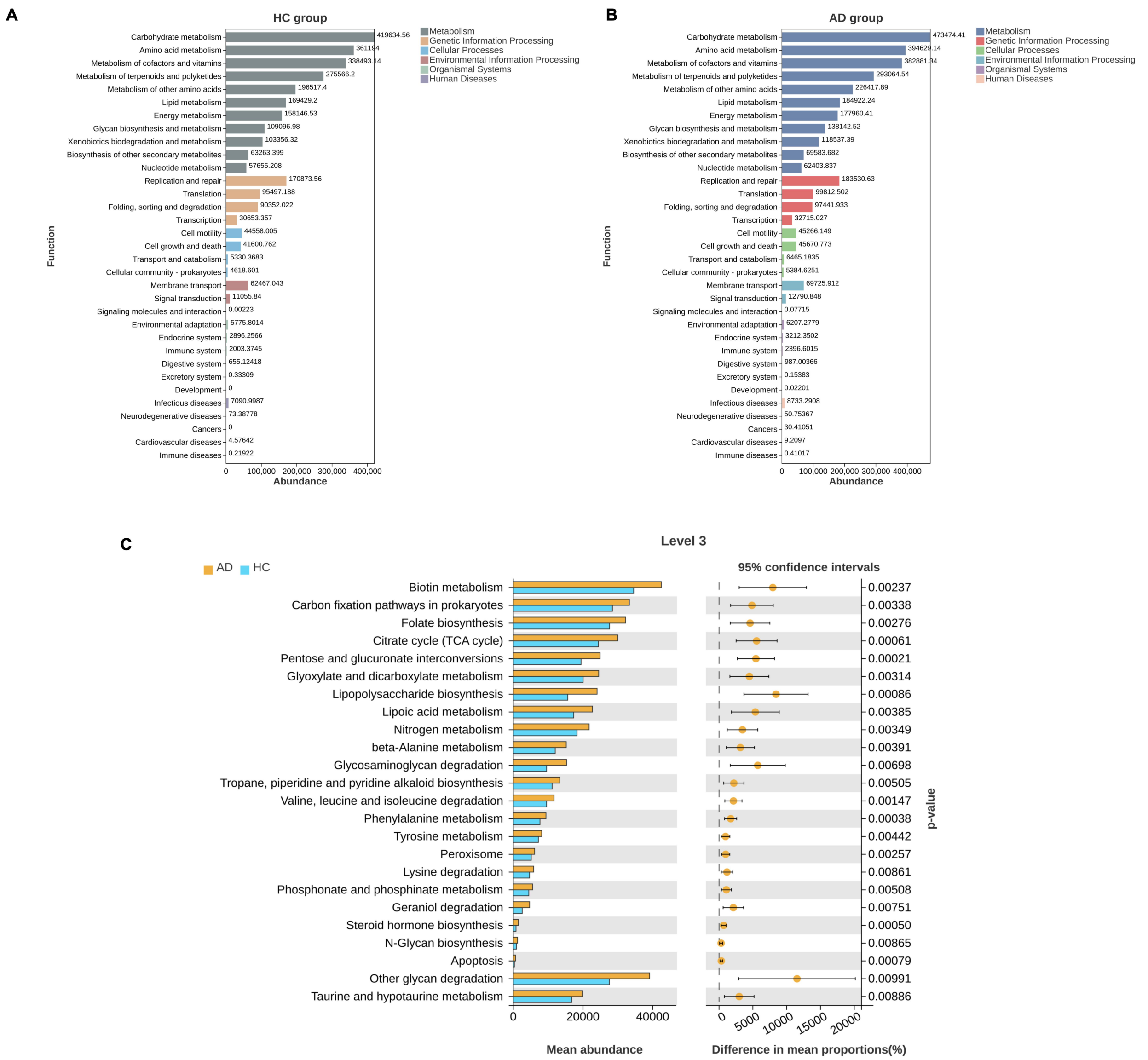

Supplementary Figure 2. Metabolic pathways of AD (A) and HC (B) based on PICRUSt2 prediction analysis. (C) Functional annotations at levels 3.

1.2 Supplementary Tables

Supplementary Table 1. Metabolites with statistical differences identified based on VIP>1 in the loading plot, FC≥1 or FC≤1, *P*<0.05 and with KEGG numbers.

Neg/pos	MS2_name	Log2_FC(case_mean/control_mean)	*P*-value	FDR	VIP	Up/Down
neg	Palmitic acid	0.563734303744753	0.00399857926590796	0.265072483583581	9.9288989227901	up

Neg/pos	MS2_name	Log2_FC(case_mean/control_mean)	*P*-value	FDR	VIP	Up/Down
				5	8	
neg	Ethylphosphonic acid	1.66563363456455	0.0291586519729495	0.59476173447388	5.60598285400145	up
neg	Stearic acid	0.746837926065453	0.01045598040789	0.4023964954304	5.24386906329759	up
neg	3-dehydrocholic acid	1.32396555951238	0.0120685612146739	0.423268394236226	4.72104210808669	up
neg	Trans-3'-hydroxycotinine o-.beta.-d-glucuronide	1.21359314061819	0.00975420416107711	0.4023964954304	3.22145598565912	up
neg	2-deoxy-d-ribose	1.9398798518114	0.0172365530314478	0.517713527919571	2.2051188190886	up
neg	4-isoxazolepropanoic acid, .alpha.-amino-2,3-dihydro-5-methyl-3-oxo-	1.64963454043365	0.0174649758945161	0.517713527919571	2.05889991884743	up
neg	Cis,cis-muconic acid	1.13699763653398	0.00113093126422403	0.168997216153285	1.97650416329519	up
neg	His-ser	1.13648506285433	0.00825515675604222	0.38733051862249	1.75569628315334	up

Neg/pos	MS2_name	Log2_FC(case_mean/control_mean)	*P*-value	FDR	VIP	Up/Down
neg	4,6-dinitroresorcinol	3.1598533232777	0.04454934490 61884	0.6690720879705 76	1.463746017566 74	up
neg	Nepodin	2.73439775253182	0.02043319681 10817	0.5329379692857 54	1.368792111851 49	up
neg	Benzoic acid, 4-[(1s)-1-[[5-chloro-2-(4-fluorophenoxy)benzoyl]amino]ethyl]-	1.32221673564127	0.00547710934 876784	0.3418410614355 15	1.337031075262 18	up
neg	Kojic acid	1.04680766283429	0.00145375062 718707	0.1777134453833 8	1.137792647530 9	up
neg	Alisol a 24-acetate	1.06723053484699	0.02660280189 20095	0.5857237366257 57	1.074641187220 37	up
neg	Thr-Pro	1.62371157887568	0.00049850018 4118341	0.1689972161532 85	1.008921116347 95	up
neg	Isovaleric acid	-0.788139089	0.02726850884 79268	0.5858044982428 59	6.411719298990 57	down
neg	18.beta.-glycyrrhetinic acid	-4.153089115	0.01950098402 15213	0.5258655182752 62	4.726599585735 26	down
neg	Thymine	-0.578304396	0.00668270965	0.345705197	3.36274717	down

Neg/pos	MS2_name	Log2_FC(case_mean/control_mean)	*P*-value	FDR	VIP	Up/Down
			15171	076278	63505	
neg	Tartaric acid	-0.914924806	0.0268748163253804	0.5857237366257 57	2.745595549295 07	down
neg	5-fluorouridine	-4.025925812	0.0418651601658422	0.6690720879705 76	2.314625629037 07	down
neg	N-(phosphonomethyl)glycine	-2.528989073	0.0391116416240959	0.6670114489787 61	1.719337365415 52	down
neg	2-methylbenzoic acid	-1.357102899	0.0402470767454186	0.6670114489787 61	1.713569202446 91	down
neg	Sinapyl alcohol	-1.438317383	0.00601605890647041	0.3418410614355 15	1.623932653227 44	down
neg	5-fluorocytidine	-1.278290251	0.0426127384039721	0.6690720879705 76	1.373604587998 56	down
neg	Mitomycin c	-1.61448782	0.018873277573435	0.5177135279195 71	1.045317092326 28	down
neg	(1e,4e)-1,5-bis(4-methoxyphenyl)penta-1,4-dien-3-one	-2.657473726	0.0421638639522507	0.6690720879705 76	1.0351812936311	down

Neg/pos	MS2_name	Log2_FC(case_mean/control_mean)	*P*-value	FDR	VIP	Up/Down
pos	Hypoxanthine	0.380383783018425	0.04968218291 20365	0.8153642675982 69	5.1226911643056 9	up
pos	Linoleoyl ethanolamide	1.52452831228344	0.00133432297 354515	0.3299113552090 38	4.3678838086744 8	up
pos	Stachydrine	1.72049648704739	0.000111419464458422	0.11019385034938	4.3515569972236 4	up
pos	3alpha,7beta,12alpha-trihydroxy-5beta-cholan-24-oic acid	0.792401857134063	0.04858692377 49796	0.8153642675982 69	4.1079121802905 4	up
pos	Theophylline	1.70694339688818	0.02441184920 78391	0.6637509174420 97	3.8466747962470 3	up
pos	(4r)-4-((3s,5s,7s,9s,10s,13r,14s,17r)-3,7-dihydroxy-10,13-dimethyl-12-oxohexadecahydro-1h-cyclopenta[a]phenanthren-17-yl)pentanoic acid	2.57737940918405	2.47550240633 76e-05	0.0489654375973578	3.5467460826049 2	up
pos	19(r)-hydroxyprostaglandin f2.alpha.	2.28001237493751	0.00202081253 110833	0.3542360163301 23	3.4653364309143 2	up

Neg/pos	MS2_name	Log2_FC(case_mean/control_mean)	*P*-value	FDR	VIP	Up/Down
pos	Fenfluramine	0.692016440757615	0.00150934316857349	0.33172008749315 1	3.35627494525882	up
pos	5-methyl-5,6-dihydrouracil	0.944545580817239	0.00691609960226958	0.4598573110348 91	3.32096955521141	up
pos	Pymetrozin	0.526743682078041	0.00662816172330664	0.4598573110348 91	3.018531712	up
pos	Cytosine	1.16805193	0.00603705910195321	0.4598573110348 91	2.67349049073063	up
pos	Phytosphingosine	0.237728271550528	0.0260005292716655	0.66980389338638 1	2.62126645658435	up
pos	7-Ketodeoxycholic acid	1.98427226501974	0.00480996458283059	0.4598573110348 91	2.29768391947234	up
pos	Acetohexamide	1.25119547255844	0.0280717046231664	0.66980389338638 1	2.16865130003399	up
pos	Linoleoylcarnitine	1.69673754725359	0.0241650260750366	0.66375091744209 7	2.15390779357773	up
pos	Guanine	1.47460210268951	0.00113200924	0.31987346936417	2.1217592838035	up

Neg/pos	MS2_name	Log2_FC(case_mean/control_mean)	*P*-value	FDR	VIP	Up/Down
			446372	7	8	
pos	Curcumin	1.444283708	0.00589084234909882	0.4598573110348911	2.106302389379355	up
pos	Lactose	0.922669662652137	0.0166271777640439	0.5820468369802788	1.988438725097144	up
pos	(r)-4-((3r,5s,8r,9s,10s,13r,14s,17r)-3-hydroxy-10,13-dimethyl-7-oxohexadecahydro-1h-cyclopenta[a]phenanthren-17-yl)pent-2-enoic acid	2.22231603830877	0.00214905571079953	0.3542360163301233	1.935970934246088	up
pos	4-quinolinecarboxylate	1.75475836287944	0.00894448114324097	0.5054909628951611	1.812621084457588	up
pos	Hydrocortisone 21-hemisuccinate	2.20342320573491	0.000296611515216176	0.1471602908309886	1.671037409432077	up
pos	.gamma.-linolenic acid	0.657673255693529	0.0339308796086733	0.71399233899953	1.572919610463433	up
pos	5-(1,2,4a,5-tetramethyl-7-oxo-3,4,8,8a-tetrahydro-2h-naphthalen-1-yl)-3-methylpentanoic	0.390252336984535	0.0337386293960671	0.71399233899953	1.459753765686833	up

Neg/pos	MS2_name	Log2_FC(case_mean/control_mean)	*P*-value	FDR	VIP	Up/Down
	acid					
pos	6-((e)-2-((1r,2s,3r,5s)-3,5-dihydroxy-2-((z)-oct-2-enyl)cyclopentyl)vinyl)tetrahydro-2h-pyran-2-one	1.022240376	0.0105906051770294	0.55400779801779 2	1.414288152181 69	up
pos	(r)-(+)-arachidonyl-1'-hydroxy-2'-propylamide	0.322369423509104	0.00250711255957132	0.356071939113079	1.407247259466 17	up
pos	Myristoyl-l-carnitine	2.03449577159358	0.0199265565470237	0.629997916809911	1.324699635	up
pos	Hydroquinone	1.036458097	0.0211761497176098	0.629997916809911	1.321531483664 82	up
pos	Tridesacetoxykhivorin	2.2394366	0.0135259289650888	0.554007798017792	1.304041053135 05	up
pos	Cyasterone	1.44899447684525	0.0150323365113461	0.582046836980278	1.258024007	up
pos	Estazolam	0.97159252074472	0.000990593027913474	0.319873469364177	1.221274081921 58	up
pos	Xanthosine	3.23307637183489	0.00830146470	0.48294991715056	1.0555247105329	up

Neg/pos	MS2_name	Log2_FC(case_mean/control_mean)	*P*-value	FDR	VIP	Up/Down
			329582	3	4	
pos	Desferrioxamine	1.41684199688093	0.00697458004602969	0.459857311034891	1.04680037293102	up
pos	Oxymatrine	1.47662063917898	0.0168585991353392	0.582046836980278	1.010593417	up
pos	Acetylcholine	-0.887611863	0.0127358229311473	0.554007798017792	15.4865899446266	down
pos	Pirimicarb-desamido	-1.279793381	0.0464064836584107	0.815364267598269	3.99538538821678	down
pos	2-(2',3',4'-trihydroxybutyl)quinoxaline	-1.237836233	0.0119960245466277	0.554007798017792	2.95349385091599	down
pos	Sulfometuron methyl	-4.227257406	0.0450394906682448	0.815364267598269	2.02518715527853	down
pos	Leukotriene f4	-2.461686988	0.0269768103273312	0.669803893386381	1.95859832853317	down
pos	Verruculotoxin	-3.609581163	0.0122027287339246	0.554007798017792	1.75546453756384	down

Neg/pos	MS2_name	Log2_FC(case_mean/control_mean)	*P*-value	FDR	VIP	Up/Down
pos	19-o-methylsiphonaxanthin	-1.658813158	0.0498045795065706	0.81536426759826 9	1.51571384484469	down
pos	Farnesol	-0.664634408	0.036828807861643	0.72537262497841 1	1.17555062787039	down
pos	7-methylguanine	-0.632556193	0.00651012916747841	0.45985731103489 1	1.12123909911545	down
